# Supplementary material for: A Novel DLLME Method Involving a Solidifying Hydrophobic Deep Eutectic Solvent Using Hydrophilic Deep Eutectic Solvent as the Dispersant for the Determination of Polychlorinated Biphenyls in Water Samples
Source: Molecules. 2024 Jul 25;29(15):3480. doi: 10.3390/molecules29153480 (PMC11314396; doi:10.3390/molecules29153480)
Supplement: Supplementary file 1 [file molecules-29-03480-s001.zip › molecules-3074891-supplementary.pdf]

Supplementary materials

for

A Novel DLLME Method Involving a Solidifying Hydrophobic Deep Eutectic Solvent Using Hydrophilic Deep Eutectic Solvent as the Dispersant for the Determination of Polychlorinated Biphenyls in Water Samples

Chunlong Peng [1,2], Shuochen Zhang [1] and Xin Li [2,3,*]

[1] Institute of Natural Medicine and Health Products, School of Pharmaceutical Sciences, Taizhou 318000, China; pengcl@tzc.edu.cn (C.P.); zhangsc0317@163.com (S.Z.)
[2] Zhejiang Provincial Key Laboratory of Plant Evolutionary Ecology and Conservation, Taizhou University, Taizhou 318000, China
[3] School of Food and Engineering, Shaanxi University of Science and Technology, Xi'an 710021, China
* Correspondence: lixin2013@tzc.edu.cn

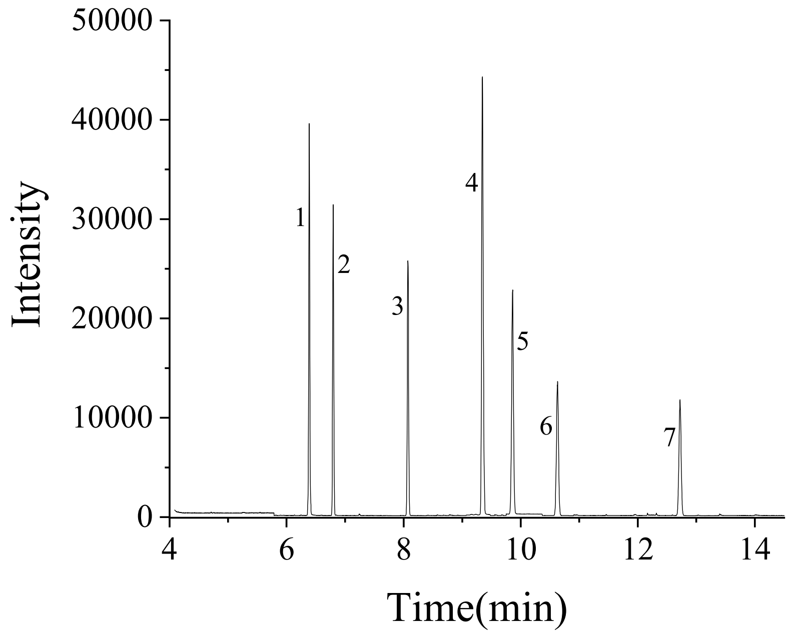

Figure S1. Total ion chromatogram of 7 indicator PCBs obtained for a 100 μg/L standard mixture. Peaks: 1) PCB28; 2) PCB52; 3) PCB101; 4) PCB118; 5) PCB153; 6) PCB138; 7) PCB180.

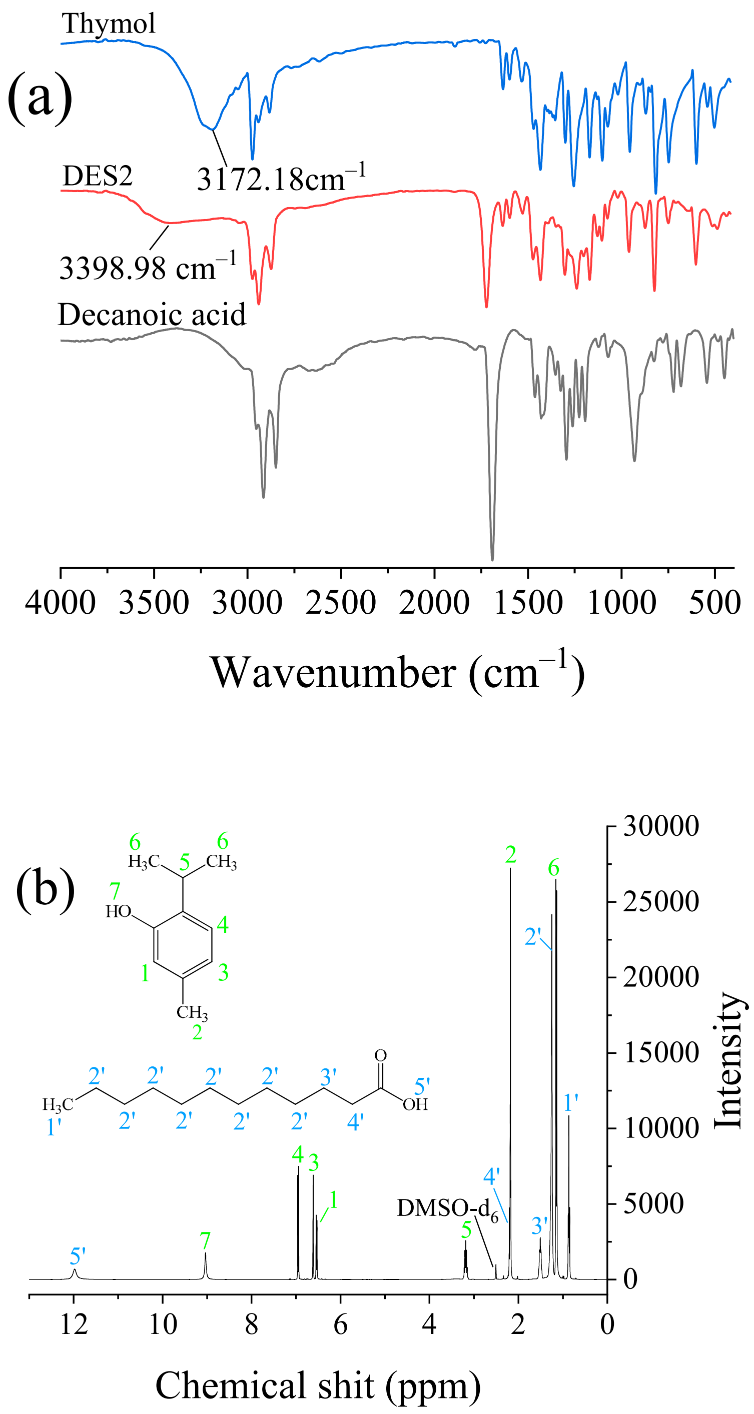

Figure S2. FT-IR spectra of thymol, decanoic acid and DES2 (a), and ^{1}H NMR spectra of DES2 (b).

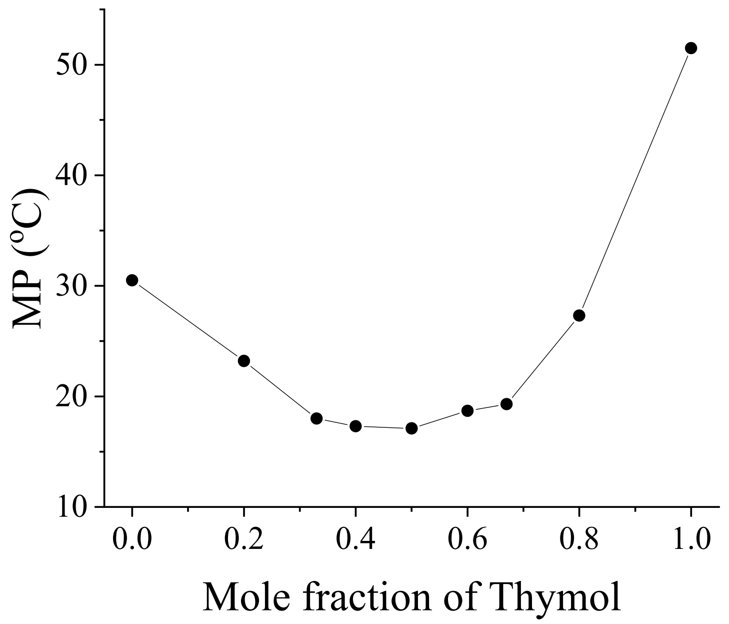

Figure S3. Solid-liquid diagram for a mixture of thymol and decanoic acid.

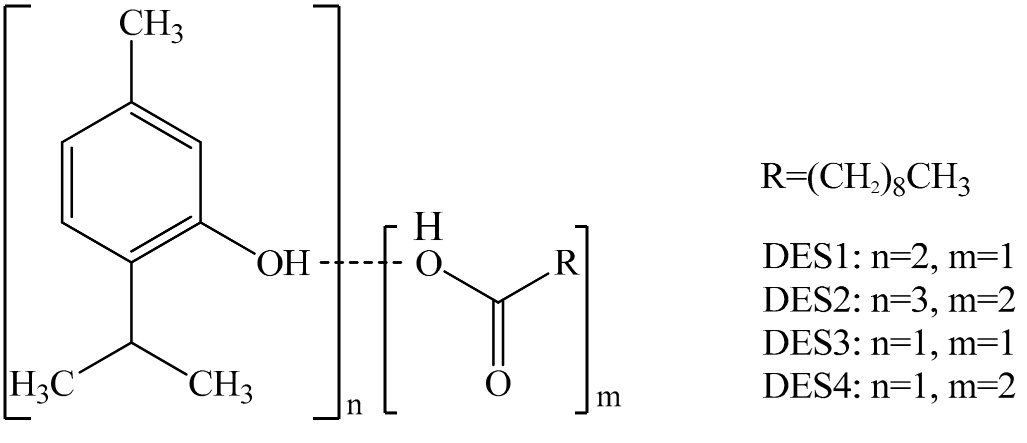

Figure S4. Structure of the prepared hydrophobic DESs.
